# Supplementary material for: Experiences of service users receiving peer support in mental health services: Qualitative findings from the international UPSIDES trial
Source: Glob Ment Health (Camb). 2026 Apr 17;13:e88. doi: 10.1017/gmh.2026.10203 (PMC13150774; doi:10.1017/gmh.2026.10203)
Supplement: Goldfarb et al. supplementary material [file S2054425126102039sup001.zip › Supplementary material file 1 - COREQ.docx]

**COREQ: 32-Item Checklist**

**Domain 1: Research Team and Reflexivity**

***Personal Characteristics***

1. **Interviewer/facilitator**: Research staff in the local sites conducted the semi-structured interviews.
2. **Credentials**: Research workers conducting the interviews had mixed backgrounds and study degrees (bachelor, master). The analysis was conducted by YG, a post-doctoral research fellow in Israel, together with AG, a PhD student who was also the research coordinator in the Israeli site, and GM, a tenured professor, site leader in Israel. In terms of positionality and reflexivity, all three coders were educated in the field of psychology and social work, and socialized in a high-income country.
3. **Occupation**: Interviewers were research workers in the UPSIDES project, analysis was carried out by the research team mentioned above.
4. **Gender**: The majority of the researchers conducting the interviews and the analysis were female.
5. **Experience and training**: The staff were guided regarding the study protocol in an online meeting with participating sites, in which sensitivity to the needs of the study population was also assured. An instructions sheet including guidelines on how to conduct the qualitative interview with the service users was presented and standard operating procedures were highlighted. The core researchers who analysed the data had several years of experience with qualitative research.

***Relationship with Participants***

1. **Relationship established**: The research staff and interviewers did not have a personal relationship with the study participants prior to commencement of the interviews. In a few occupations, there was a former general acquaintance as part of the research team.
2. **Participant knowledge of the interviewer**: What did the participants know about the researcher? (e.g., personal goals, reasons for doing the research): In all study sites the interviewers introduced themselves, the study and the study aim (according to the topic guide guidelines) before the beginning of the interview.
3. **Interviewer characteristics**: What characteristics were reported about the interviewer/facilitator? (e.g., biases, assumptions, reasons, and interests in the research topic): Interviewer characteristics other the those reported in the topic guide were not shared with the participants.

**Domain 2: Study Design**

***Theoretical Framework***

1. **Methodological orientation and theory**: What methodological orientation was stated to underpin the study? Thematic content analysis.

***Participant Selection***

1. **Sampling**: How were participants selected? Following the intervention, participants were purposively selected at each site to capture a broad spectrum of responses, including strongly positive and negative experiences. Based on pre-post intervention change scores on quantitative measures of social inclusion (Social Inclusion Scale) and personal recovery in psychiatric services (Brief INSPIRE), they were grouped into ‘low responders’ who presented the least benefit from the intervention, and ‘high responders’ who benefited the most. The top and bottom 20% of the combined ranking were randomly selected and approached until a sufficient number of participants from each site were interviewed (pre-determined to be between 6-8), or when all listed participants were approached.
2. **Method of approach**: How were participants approached? Participants were invited to participate via phone calls in which general information about the purpose of the study was given. If participants agreed, a specific date and venue were scheduled.
3. **Sample size**: How many participants were in the study? 33 participants.
4. **Non-participation**: Documented reasons for not participating were a decline in mental or physical health, difficulties attaining consent due to mental illness, and geographical relocation.

***Setting***

1. **Setting of data collection**: interviews were conducted face-to-face in various locations agreed upon by the interviewer and the participant, mostly research center or mental health service facility.
2. **Presence of non-participants**: Was anyone else present besides the participants and researchers? No.
3. **Description of sample**: See Table 1 for participant information.

***Data Collection***

1. **Interview guide**: Were questions, prompts, guides provided by the authors?
   Semi-structured topic guide was used. Topics captured everyday experiences with peer support, subjective appraisal of the positive/negative effects of peer support and attitudes towards provision of peer support within mental health settings. The topic guide was developed by a core research team, to address all aspects of the interventions emphasizing clear questions and addressing current issues (e.g. the corona pandemic).
2. **Repeat interviews**: Repeat interviews were not carried out.
3. **Audio/visual recording**: Audio recordings only**.**
4. **Field notes**: Notes were taken by the interviewers immediately after, mentioning key themes, important insights, the tone of the interviewee, etc.
5. **Duration**: Duration of the interviews was between about 30 to 70 minutes.
6. **Data saturation**: The sample size was predetermined by the research design described above.
7. **Transcripts returned**: Were transcripts returned to participants for comment and/or correction?
   No

**Domain 3: Analysis and Findings**

***Data Analysis***

1. **Number of data coders**: How many data coders coded the data? Three coders
2. **Description of the coding tree**: Four themes describing service user experience of the UPSIDES peer-support intervention were identified and described in the results section of the paper, with example quotes.
3. **Derivation of themes**: Themes were identified during the data analysis process.
4. **Software**: MAXQDA 24 software was used to manage and analyze the data.
5. **Participant checking**: Did participants provide feedback on the findings? No

***Reporting***

1. **Quotations presented**: Participant quotations are presented within the results section of the paper, and additional quotes are provided in Table 2. For each participant the site, code, age and gender were mentioned.
2. **Data and findings consistent**: Was there consistency between the data presented and the findings? Yes.
3. **Clarity of major themes**: The four themes are clearly presented in the results section of the paper.
4. **Clarity of minor themes**: While sub-themes were not derived from the data, subtleties in the themes and differences between sites are clearly mentioned and discussed within the results section and the discussion.
