# Supplementary material for: Experiences of service users receiving peer support in mental health services: Qualitative findings from the international UPSIDES trial
Source: Glob Ment Health (Camb). 2026 Apr 17;13:e88. doi: 10.1017/gmh.2026.10203 (PMC13150774; doi:10.1017/gmh.2026.10203)
Supplement: Goldfarb et al. supplementary material [file S2054425126102039sup001.zip › Supplementary file 2 - context of the study sites.docx]

**Supplementary material 2: context of the study sites**

| **City, Country** | Ulm/  Guenzburg, Germany  (Ulm) | Hamburg,  Germany  (UKE) | Butabika, Uganda  (BU) | Dar es Salaam, Tanzania  (DS) | Be’er Sheva,  Israel  (BGU) | Pune, India  (PU) |
| --- | --- | --- | --- | --- | --- | --- |
| **Income level** | High-income | High-income | Low-income | Low-income | High-income | Lower-middle-income |
| **Implementation at/in** ^[1]^ | Catchment area of Ulm  University’s Department of Psychiatry and  Psychotherapy II; District Hospital Ulm/ Guenzburg | Catchment area of University Medical Centre Hamburg-Eppendorf and  community  services | Butabika Hospital, Kampala | Muhimbili  National Hospital at the Department of Psychiatry  and Mental Health | Kidum Proyektim Shikumim (supported residential and mentoring services); Enosh (Israeli Mental Health Association) | Hospital for Mental  Health in Ahmedabad, Gujarat |
| **PSW involved prior to UPSIDES**  ^[1]^ | No/little | Yes | Yes | No | Yes | Yes |
| **MH services in which UPSIDES PSWs are involved in*** | Outpatient  services | Outpatient services | In- and outpatient  services | In- and outpatient  services | Outpatient  services | Inpatient services |
| **Area of UPSIDES peer support*** | Rural area | Urban area | Mixed area | Urban area | Urban area | Urban area |

*Information provided by research workers at study sites

**References**

1. Moran GS, Kalha J, Mueller-Stierlin AS, Kilian R, Krumm S, Slade M, et al. Peer support for people with severe mental illness versus usual care in high-, middle- and low-income countries: study protocol for a pragmatic, multicentre, randomised controlled trial (UPSIDES-RCT). Trials. 2020;21:371. doi:10.1186/s13063-020-4177-7.
