# Supplementary material for: Experiences of service users receiving peer support in mental health services: Qualitative findings from the international UPSIDES trial
Source: Glob Ment Health (Camb). 2026 Apr 17;13:e88. doi: 10.1017/gmh.2026.10203 (PMC13150774; doi:10.1017/gmh.2026.10203)
Supplement: Goldfarb et al. supplementary material [file S2054425126102039sup001.zip › Supplementary file 3 - interview topic guide.docx]

# Supplementary material 3: Topic Guide for Service User Interviews

The topic guide includes five topics, which SUs are be asked about in the interviews. For each topic there is a key question, followed by additional encouragement questions, asked if more prompting is needed. Interviewers are guided to: use non-judgmental probing to help participants elaborate on answers; use open questions to introduce a new topic; use clarifying questions to probe a topic; at the end of the interview thank the participants for
their contribution.

**Interview introduction**: The interviewer welcomes the participant, introduces themselves, and explains that the meeting is part of the UPSIDES project, which explores the impact of peer support for people with mental health difficulties. The participant is informed that the aim is to understand their personal experiences—both positive and negative—of receiving peer support.

**Interview content**: the topic guide includes five main themes, each with key questions and prompts to guide a flexible but consistent interview process across sites.

## 1. Experiences

- What did UPSIDES involve for you (e.g., number of meetings, locations, timing)?
- What made the UPSIDES peer support intervention unique or different from other services you've received?
- What did you like most and least about the peer support you received?

## 2. Appraisal

- How did peer support impact your self-esteem, self-care, mental health management, and social life?
- What were the positive and negative effects on your recovery?
- Was there anything unique about peer support that contributed to these effects?

## 3. Contextual Factors

- What helped you participate in UPSIDES (e.g., location, people, supports)?
- What challenges did you face in attending or staying involved?
- Describe any barriers or facilitators you experienced.

## 4. COVID-19

- How did the COVID-19 pandemic affect your life and your experience with peer support?

## 5. Closing Reflections

- Is there anything else you’d like to share about your experience?
